# Supplementary material for: Mechanically tunable organogels from highly charged polyoxometalate clusters loaded with fluorescent dyes
Source: Nat Commun. 2023 Dec 14;14:8327. doi: 10.1038/s41467-023-43989-3 (PMC10721816; doi:10.1038/s41467-023-43989-3)
Supplement: Supplementary file 1 — Supplementary Information [file 41467_2023_43989_MOESM1_ESM.pdf]

Supplementary Information

Mechanically tunable organogels from highly charged polyoxometalate clusters loaded with fluorescent dyes

Fenghua Zhang[1], Zhong Li[1]*, Xun Wang[1]*

[1]Engineering Research Center of Advanced Rare Earth Materials, Department of Chemistry, Tsinghua University, Beijing, China.

*E-mail: zhongli@mail.tsinghua.edu.cn; wangxun@mail.tsinghua.edu.cn

Table of Contents

1. **Supplementary Notes 1-3** ········· **3**

2. **Supplementary Figures 1-31** ········· **5**

3. **Supplementary Tables 1-7** ········· **36**

4. **Supplementary References 1-7** ········· **43**

1. Supplementary Notes

Supplementary Note 1. Oleylamine ligand density on Ca_2-P_2W_{16} NWs

We have performed TGA measurements for Ca-PW_{12} NWs and Ca_2-P_2W_{16} NWs samples to determine the alkyl chain density on the surfaces. First, the density of oleylamine coated on the surface is assumed to be even-distributed. We obtained the amount the oleylamine coated on NWs when they are burning out at elevated temperatures (> 450 ºC). From the TGA curve in Supplementary Fig. 4, one can find a continuous weight loss of ≈ 46% in the range of 25-450 ºC, which includes three main processes: (1) Loss of adsorbed water and organic solvent in the range of 25-250 ºC, which is determined to be ≈ 24%; (2) In the range of 250-450 ºC, which is also accompanied by the burning of organic ligands, which comprises of ≈ 22%. Hence the surface ligand density can be calculated to be ≈ 2 oleylamine per Ca-PW_{12} and ≈ 6 oleylamine per Ca_2-P_2W_{16}.

Supplementary Note 2. The interaction energy of organic liquid-NW and NW-NW

For the modeling of the interaction energy between NWs and n-octane, the main attractive force is the van der Waals force from the chain-chain interactions. Assuming that two mutually parallel alkyl chains have maximized van der Waals force, as indicated by Salem, the attractive interactions between NWs and solvent can be estimated from the pairs of alkyl chains that are parallel arranged. For simplicity, the oleylamine (C_{18}) alkyl chain grafting density for both NWs and solvent (n-octane) is 6 chains nm^{-2}.

The interaction energy between NWs and NWs. The interaction energy between NWs and NWs can be estimated by the most reasonable model, which assumes that the ligands lie on the surface of NW (Supplementary Fig. 5). To the first assumption, the interaction energy can be estimated by two parallel C18 chains. The van der Waals attraction (U_{C18}) between two nearest parallel alkyl chains of length L from N identical basic units ($L=N\lambda$) and separated by a distance D has been given by Salem:[1]

$$U_{\mathrm{C18}} = A\frac{3\pi}{8\lambda^2}\frac{L}{D^5} \quad (1)$$

Where A is the Hamaker constant of methylene units. With Salem's conclusion that the attractive energy is correlated with the length of the alkyl chain, and with the attractive energy for C18 is calculated to be -14.4 $\mathrm{k_B}T$ per molecule, we could relate the overlapping length and interaction strength:

$$U_{\mathrm{attr}} \approx (-7.2\mathrm{k_B}T) \times (2L - d) \quad (2)$$

Where d is the distance between two NWs. The elastic repulsion energy between two C18 chains can be calculated based on the elastic modulus (E), which is ≈ 1.3 GPa for octadecyl alkyl chains.[2] Hence the elastic repulsion energy can be estimated to be:

$$U_{\mathrm{el}} \approx \frac{1}{2} \times \frac{EA_0}{L} \approx (25.8\mathrm{k_B}T) \times (2L - d)^2 \quad (3)$$

Where A_0 is the cross-sectional area of an alkyl chain ($A_0 \approx 0.25$ nm^2). Considering the attractive and elastic repulsive energy between two parallel C18 chains, we claim that the overall contribution from the ligand-ligand of NWs interactions can be about -$\mathrm{k_B}T$. **The interaction energy between NWs and organic solvent.** In this case, we found that nearly all the alkyl chains between NWs and solvent (n-octane). Oleylamine and n-octane are parallel when they are approaching each other. Hence the total energy between the surface of NWs can be estimated from the following equation:

$$U_{\mathrm{NWs-octane}} \approx S\rho_{C8}[(-4.0\mathrm{k_B}T) \times (2L - d) + (14.3\mathrm{k_B}T) \times (2L - d)^2] \quad (4)$$

Where S is the total contacting areas between the NW and organic liquid molecules. With this simple model, the interaction energy between NWs and organic liquid can be tens of $\mathrm{k_B}T$, one or two orders of magnitude larger than that interaction energy between NWs and NWs.

Supplementary Note 3. The composition of fracture toughness of NW-based organogels [3]

For soft materials with large energy dissipation (e.g., organogels), the fracture toughness can be decomposed into intrinsic fracture toughness Γ_0 and the contribution Γ_d of bulk dissipation to fracture toughness $\Gamma = \Gamma_0 + \Gamma_d$. The intrinsic fracture toughness Γ_0 is the energy required to break the chemical bond during the fracture of the material. Therefore, we believe that the fracture toughness of organogels is mainly due to the contribution of energy dissipation in the larger damage (i.e., mainly including entanglement between the NWs and the interaction between the NWs and organic liquids). The NW-based organogel system basically does not contribute to intrinsic fracture toughness, that is, the contribution of Γ_0 to Γ can be ignored.

2. Supplementary Figures

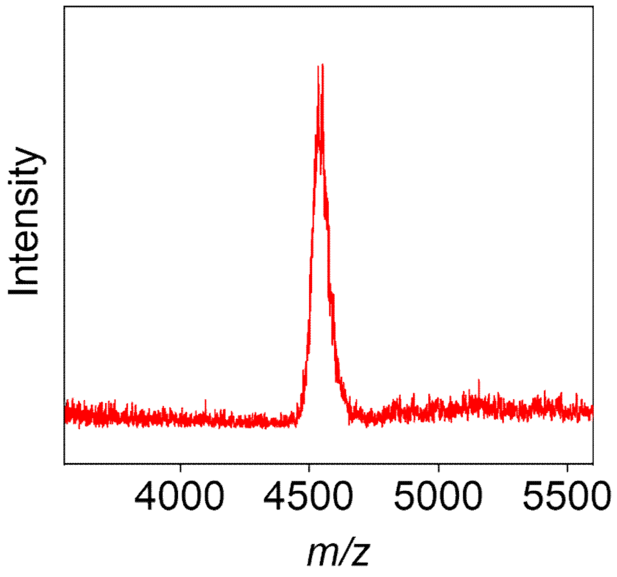

Supplementary Fig. 1 Matrix-assisted laser desorption ionization time-of-flight mass spectrometry (MALDI-TOF-MS) of $K_6P_2W_{18}O_{62}$•$14H_2O$.

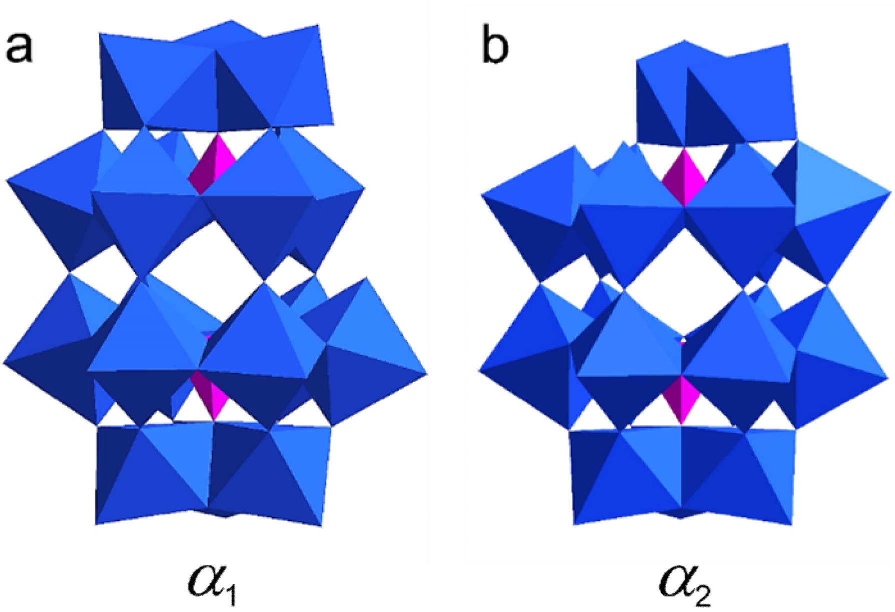

Supplementary Fig. 2 Polyhedral representations of two monovacant Dawson-type POM isomers α_1 (**a**) and α_2 (**b**).

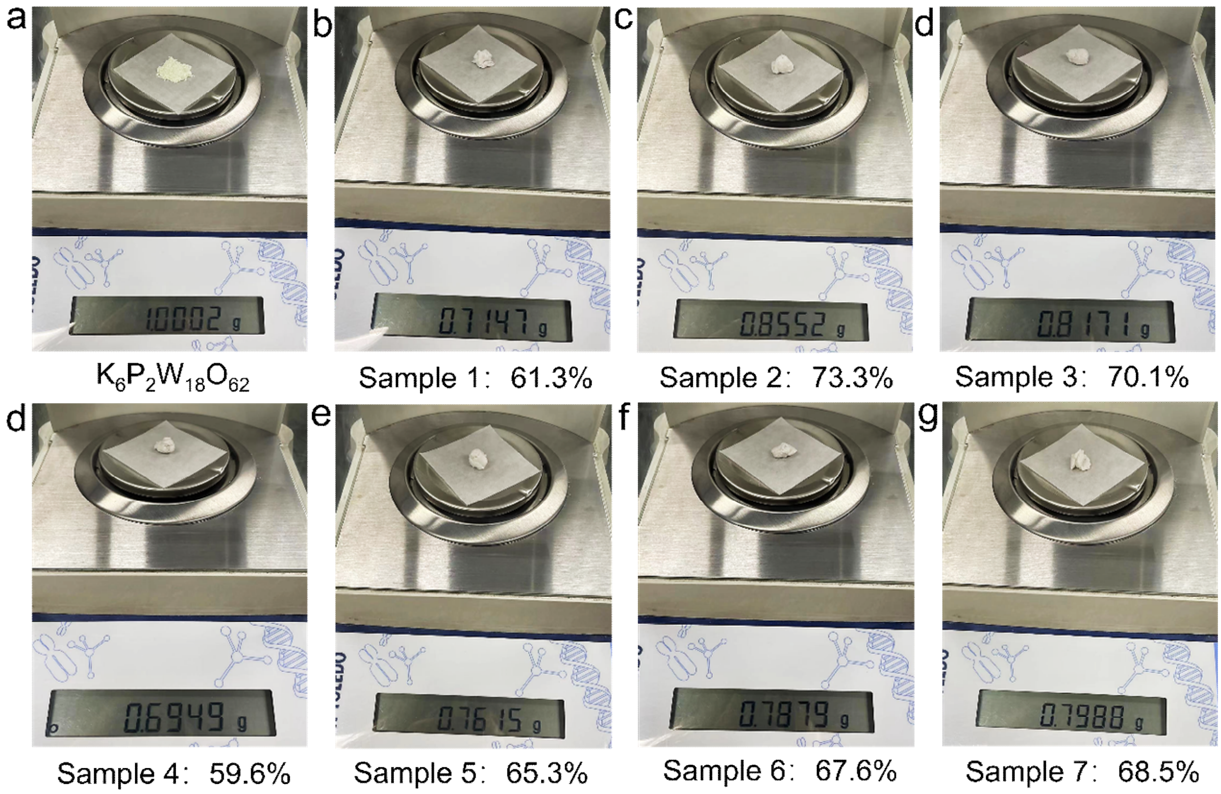

Supplementary Fig. 3 Yields of NWs. Photographs of $K_6P_2W_{18}O_{62}\cdot 14H_2O$ powder (**a**), Ca_2-P_2W_{16} NWs solid (**b-g**). Yields are falling between 73.3% and 59.6%.

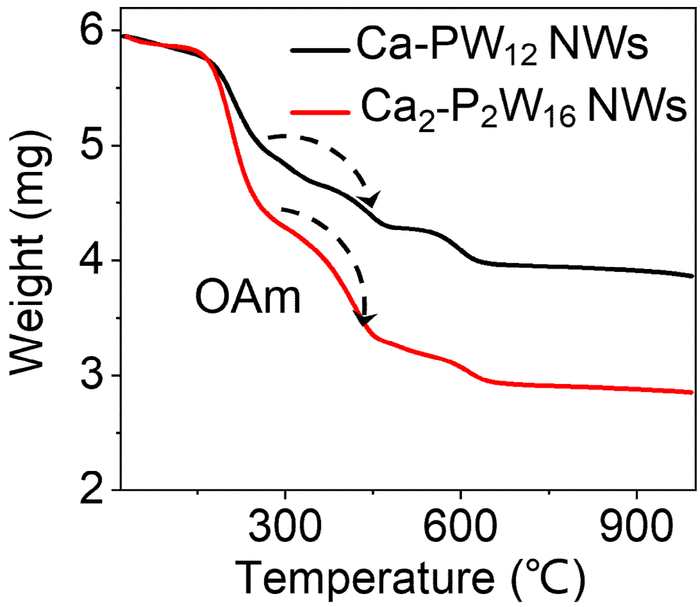

Supplementary Fig. 4 Thermogravimetric analysis on Ca-PW_{12} NWs and Ca_2-P_2W_{16} NWs.

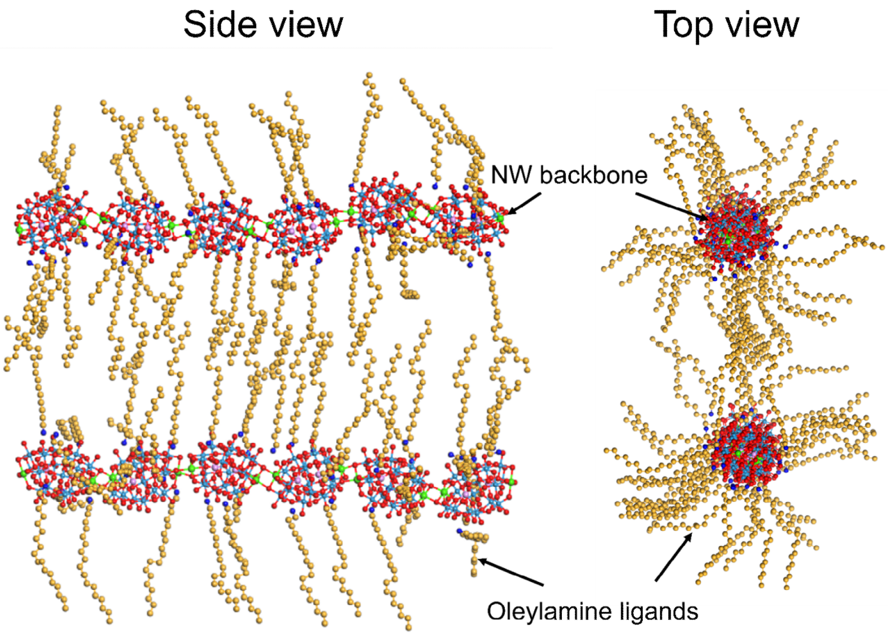

Supplementary Fig. 5 Schematic of the interaction between Ca_2-P_2W_{16} NWs and Ca_2-P_2W_{16} NWs.

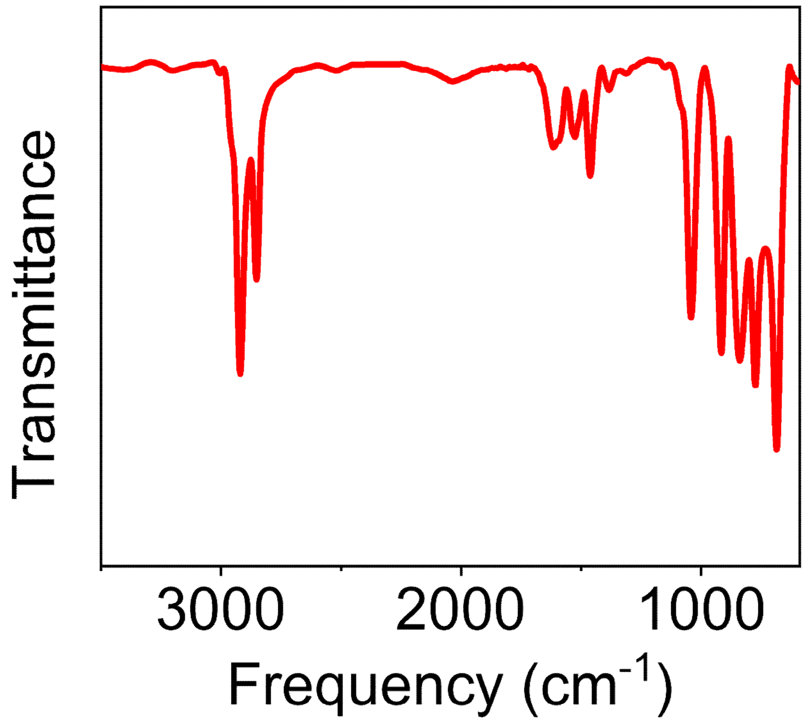

Supplementary Fig. 6 FTIR spectroscopy of Ca_2-P_2W_{16} NWs.

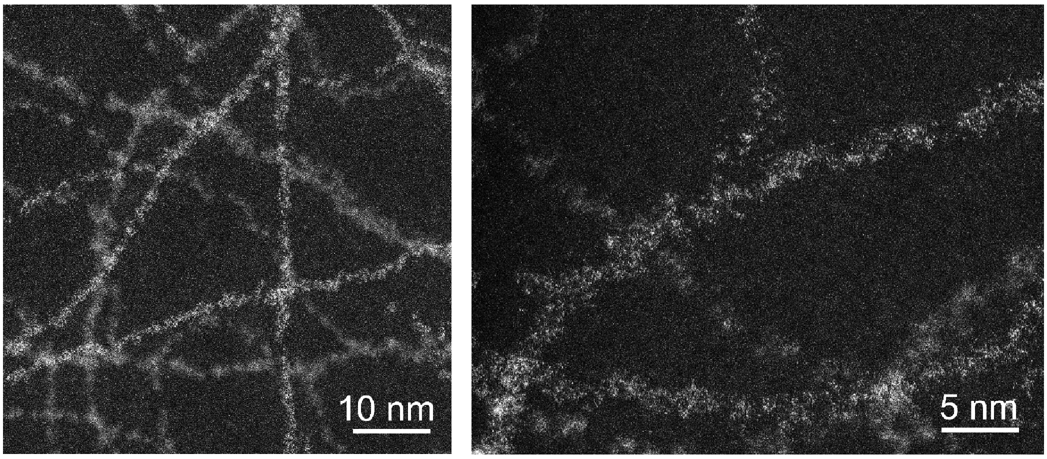

Supplementary Fig. 7 The atomic-resolution AC high-angle annular-dark field scanning TEM (AC-HAADF-STEM) images of Ca_2-P_2W_{16} NWs at different scales.

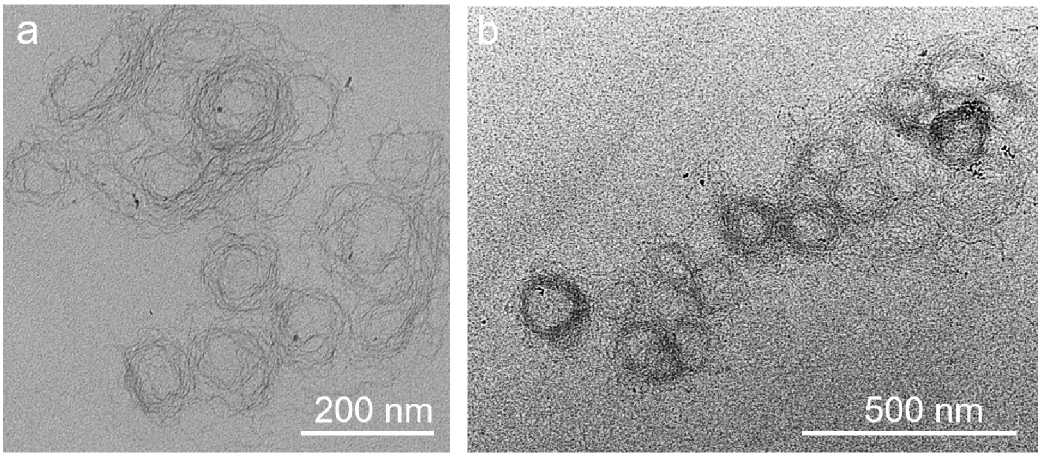

Supplementary Fig. 8 TEM images of coiled Ca_2-P_2W_{16} NWs at different scales.

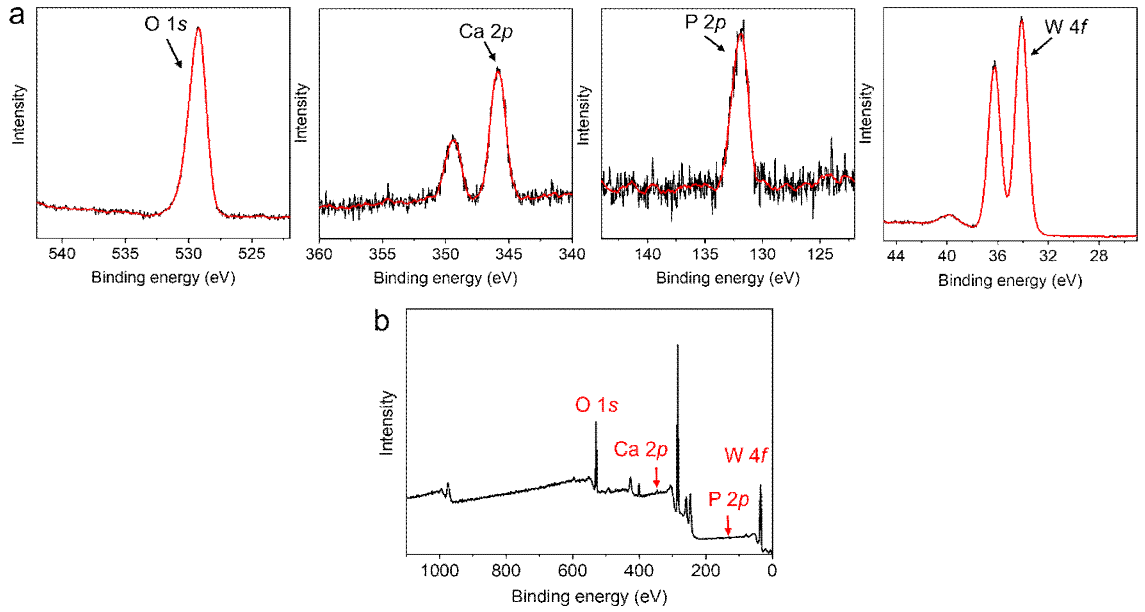

Supplementary Fig. 9 XPS spectra of Ca_2-P_2W_{16} NWs. a XPS spectra of O 1*s*, Ca 2*p*, P 2*p*, and W 4*f*. **b** Full XPS spectra of Ca_2-P_2W_{16} NWs.

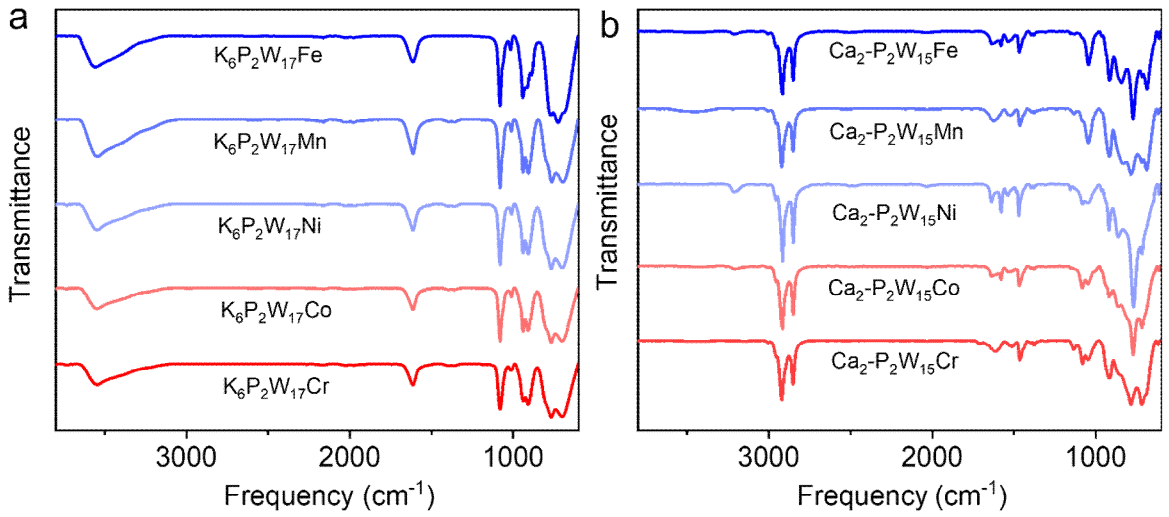

Supplementary Fig. 10 FTIR spectroscopy of $K_xP_2W_{17}MO_{61}$ (M=Fe, Mn, Ni, Co, Cr) (**a**) and Ca-$P_2W_{15}M$ (M=Fe, Mn, Ni, Co, Cr) NWs (**b**).

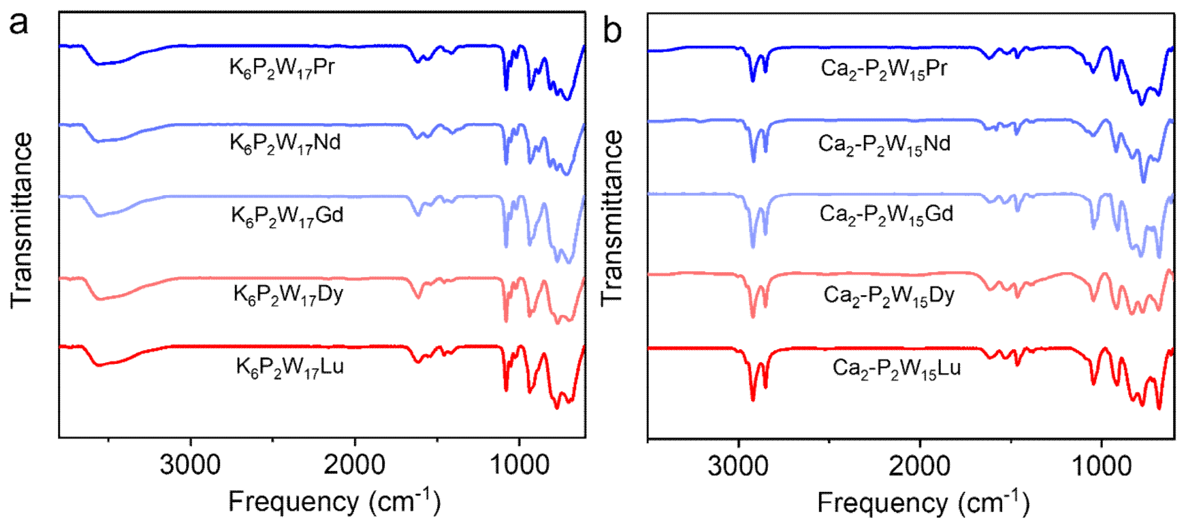

Supplementary Fig. 11 FTIR spectroscopy of $K_6P_2W_{17}MO_{61}$ (M=Pr, Nd, Gd, Dy, Lu) (**a**) and Ca-P_2W_{15}M (M=Pr, Nd, Gd, Dy, Lu) NWs (**b**).

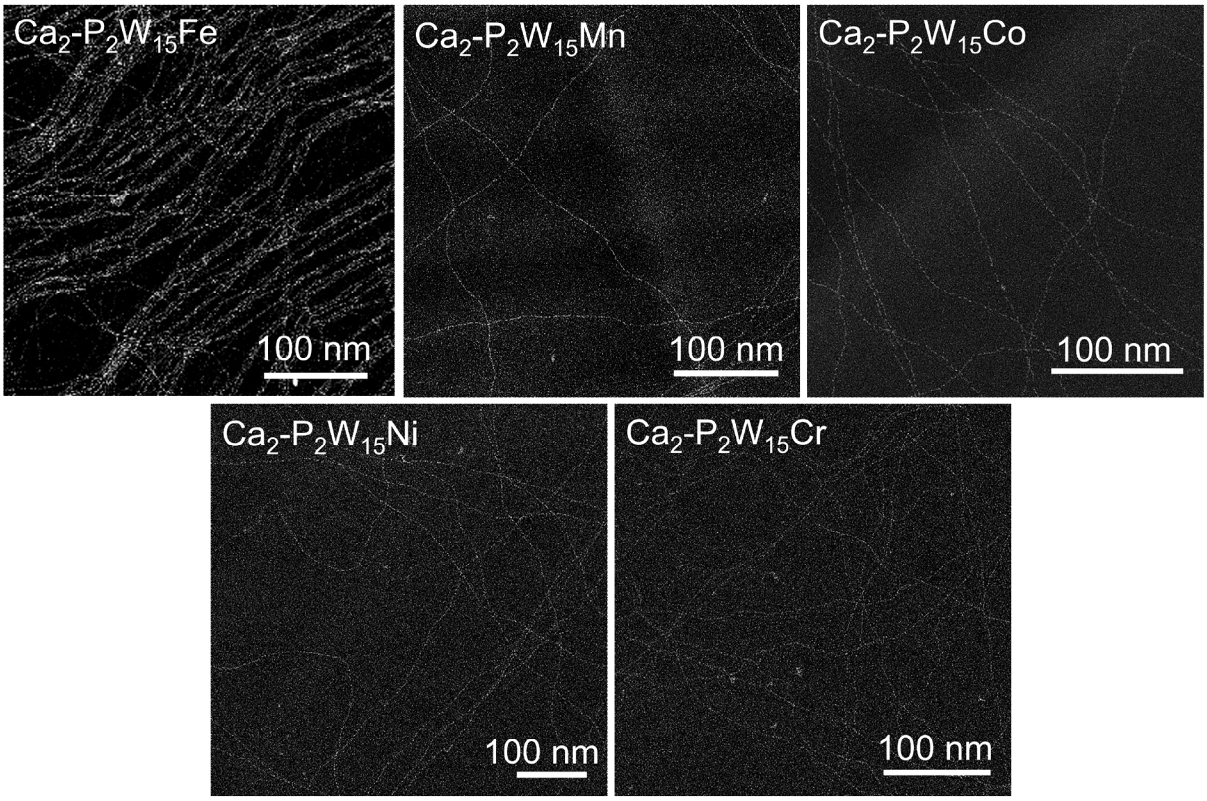

Supplementary Fig. 12 STEM images of Ca_2-$P_2W_{15}M$ (M=Fe, Mn, Ni, Co, Cr) NWs.

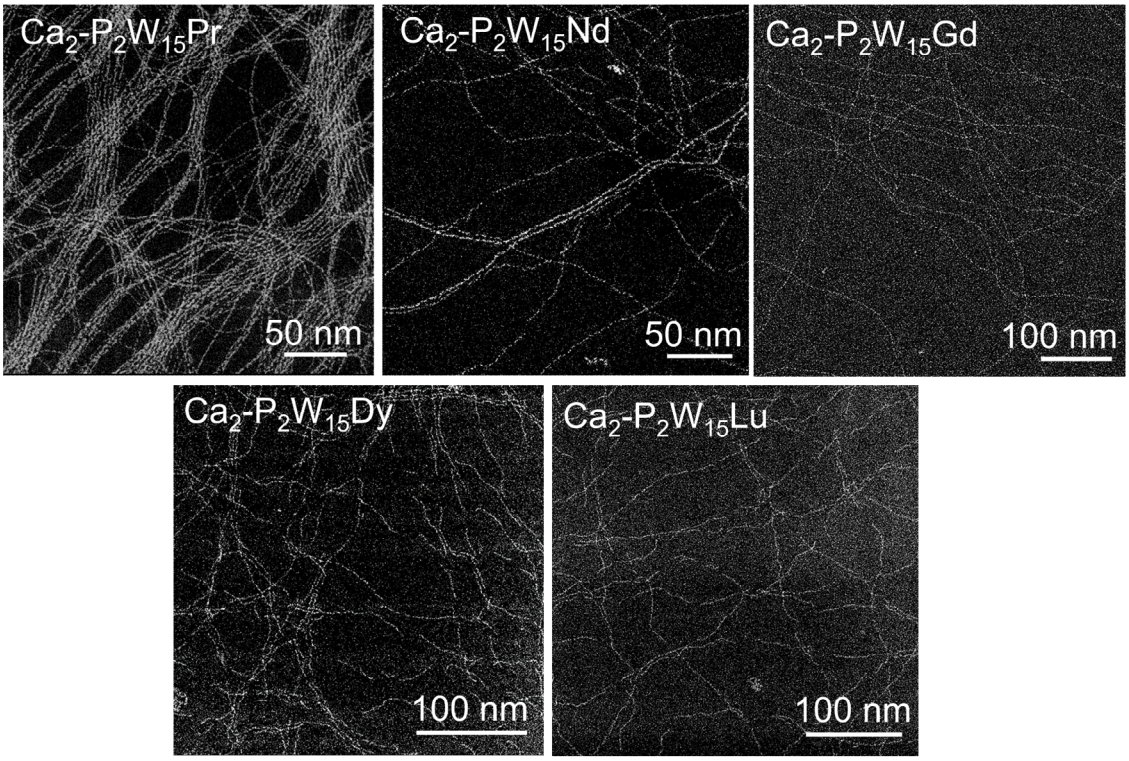

Supplementary Fig. 13 STEM images of Ca_2-$P_2W_{15}M$ (M=Pr, Nd, Gd, Dy, Lu) NWs.

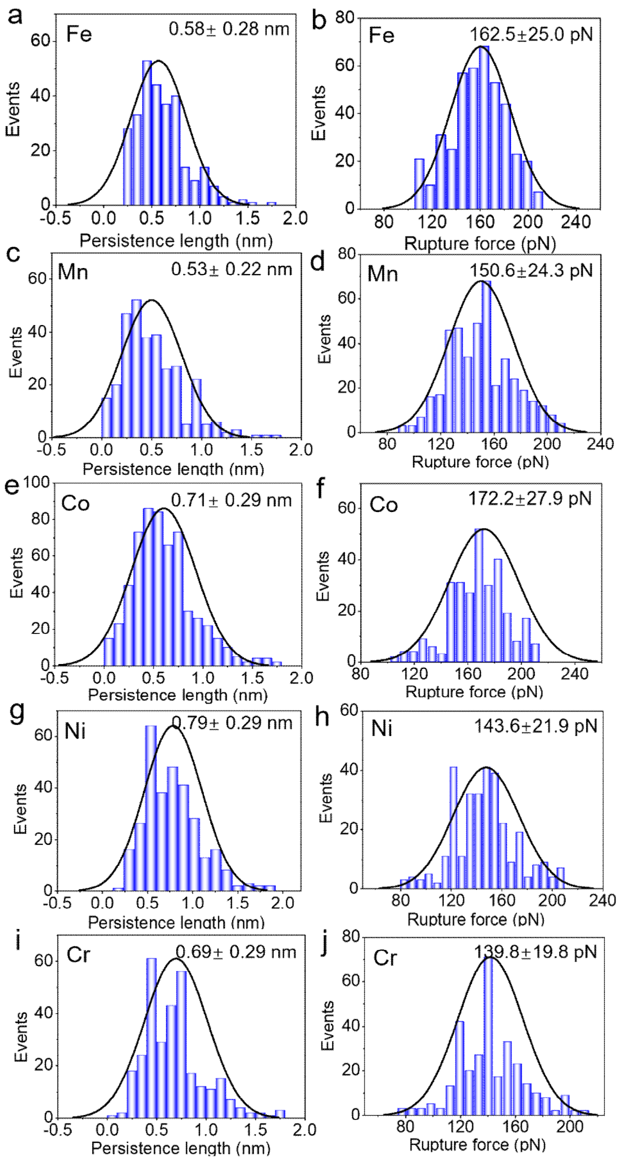

Supplementary Fig. 14 Statistical histograms of persistence lengths and rupture forces of Ca_2-$P_2W_{15}M$ (M=Fe, Mn, Ni, Co, Cr) NWs. These statistical histograms were obtained from representative samples of 300, respectively.

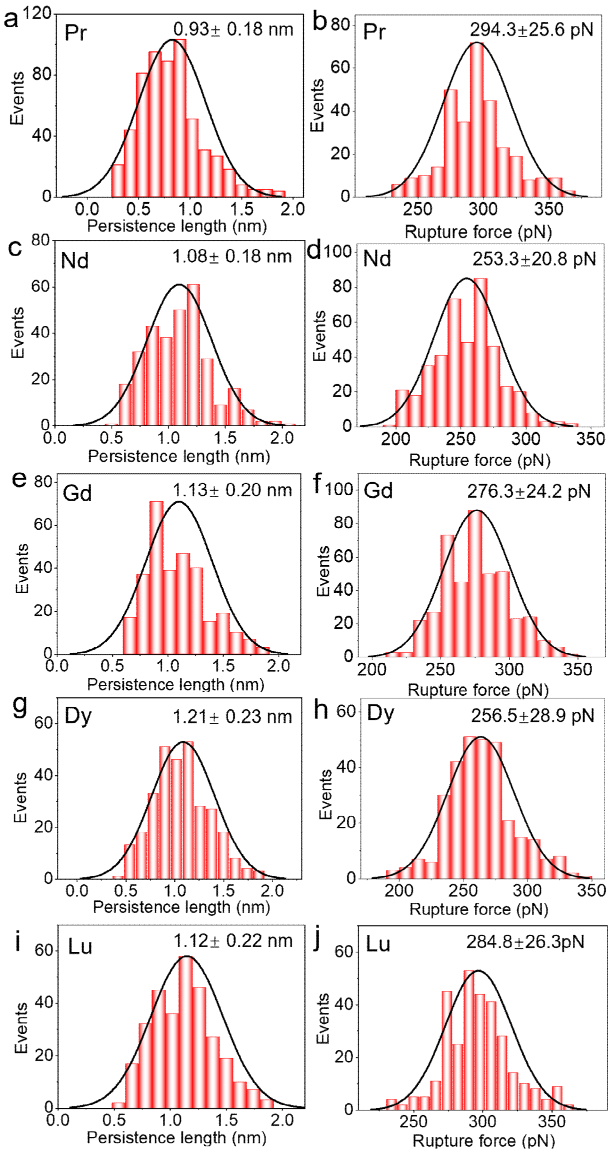

Supplementary Fig. 15 Statistical histograms of persistence lengths and rupture forces of Ca_2-$P_2W_{15}M$ (M=Pr, Nd, Gd, Dy, Lu) NWs. These statistical histograms were obtained from representative samples of 300, respectively.

The persistence lengths of Ca_2-$P_2W_{15}TM$ NWs and Ca_2-P_2W_{15} RE NWs are 0.53-0.79 nm and 0.93-1.21 nm, respectively. Thus, the flexibilities of Ca_2-P_2W_{16} and Ca_2-$P_2W_{15}M$ NWs are generally lower than that of Ca-PW_{12} NWs. In addition, we found the rupture forces of Ca_2-$P_2W_{15}RE$ NWs (253.3-294.3 pN) are greater than that of Ca_2-P_2W_{16} NWs and Ca_2-$P_2W_{15}TM$ NWs.

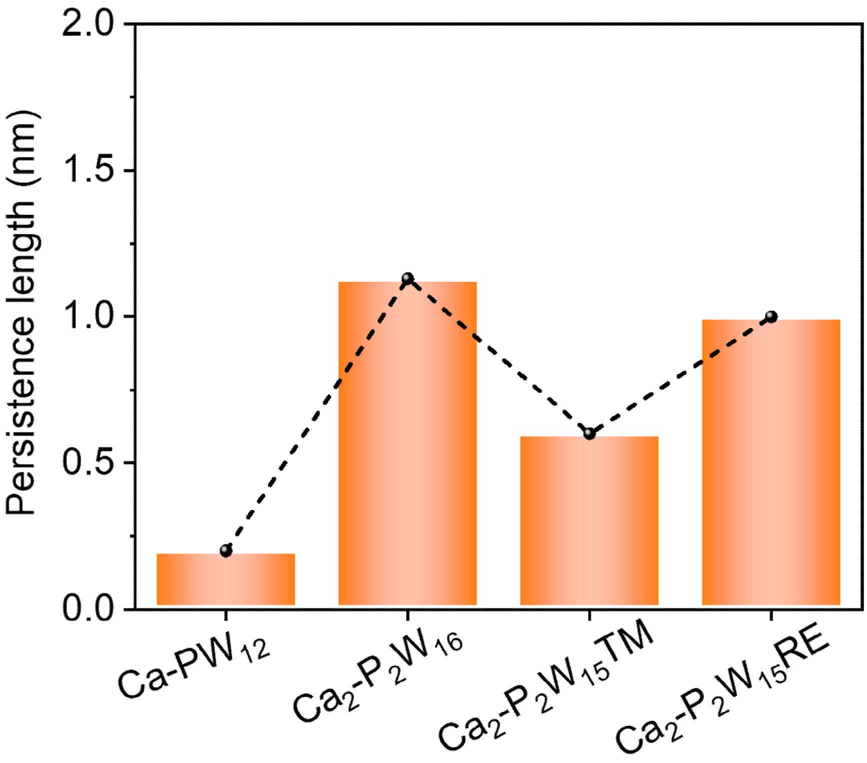

Supplementary Fig. 16 Histogram of the persistence lengths of NWs, namely Ca-PW_{12}, Ca_2-P_2W_{16}, and Ca_2-$P_2W_{15}M$ (M=TM, RE) NWs.

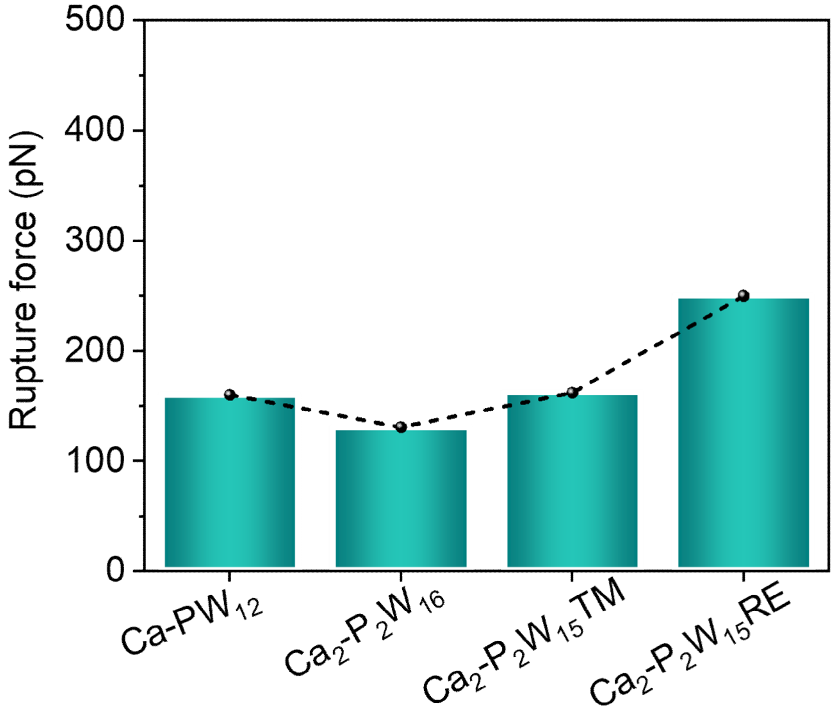

Supplementary Fig. 17 Histogram of rupture forces of Ca-PW_{12}, Ca_2-P_2W_{16}, and Ca_2-$P_2W_{15}M$ (M=TM, RE) NWs.

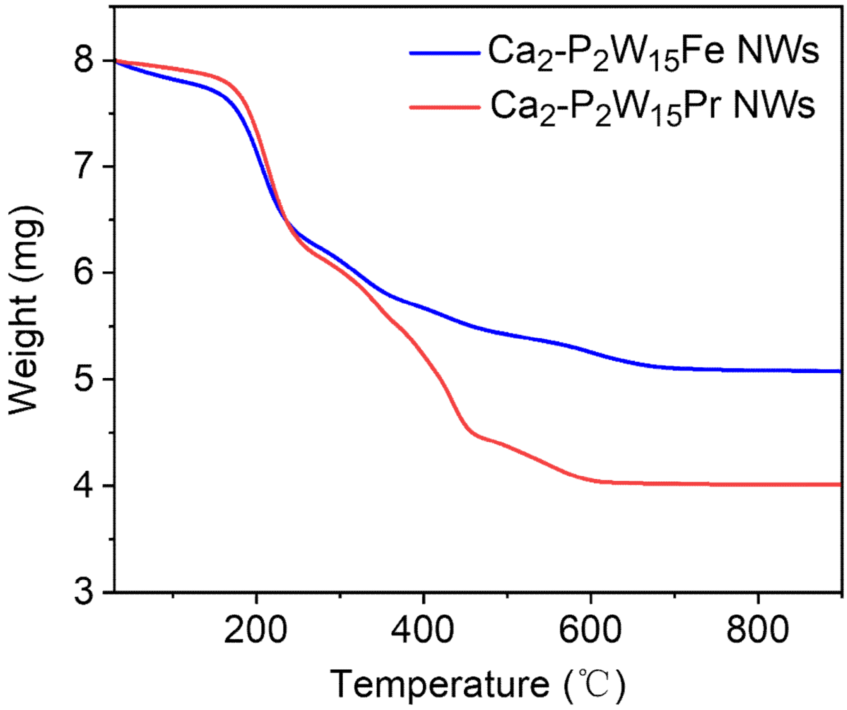

Supplementary Fig. 18 TGA of Ca_2-P_2W_{15}Fe NWs and Ca_2-P_2W_{15}Pr NWs. The surface oleylamine density can be calculated to be ≈ 5 per Ca_2-P_2W_{15}Fe and ≈ 7 oleylamine per Ca_2-P_2W_{15}Pr.

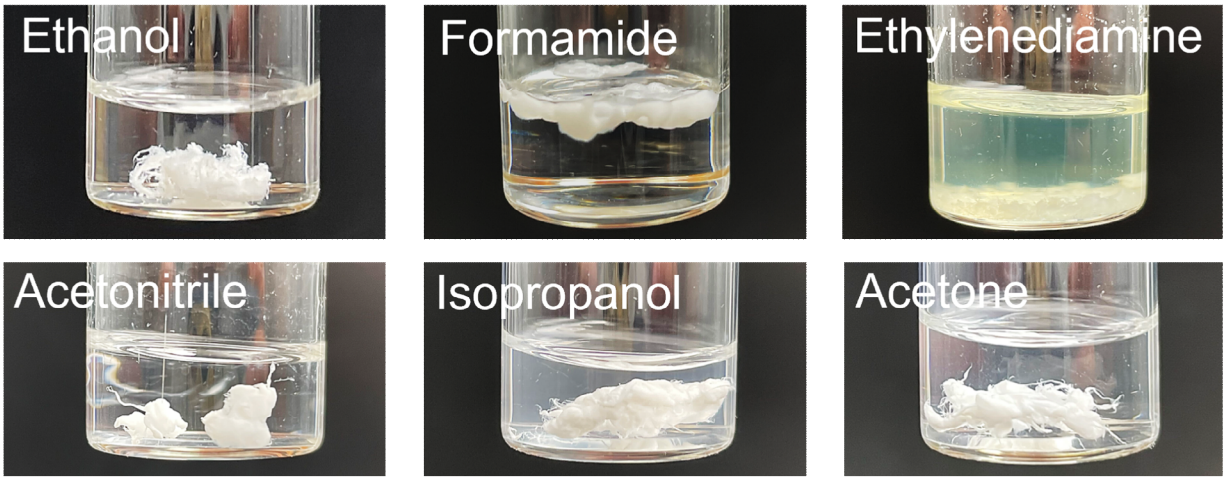

Supplementary Fig. 19 Photographs of Ca_2-P_2W_{16} NWs in organic liquids.

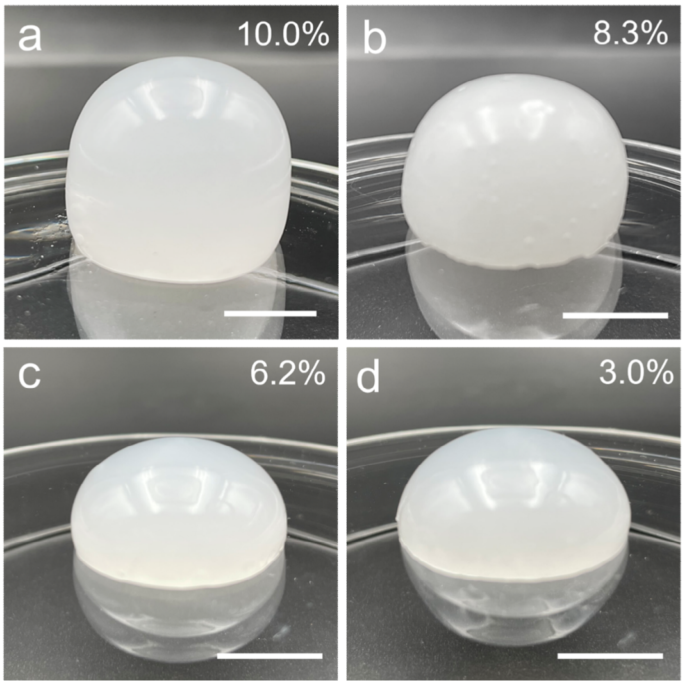

Supplementary Fig. 20 Photographs of Ca_2-P_2W_{16} NW-octane gels with different mass fractions of NWs. (**a**) 10.0%, (**b**) 8.3%, (**b**) 6.2% and (**c**) 3.0%. Scale bars are 1 cm.

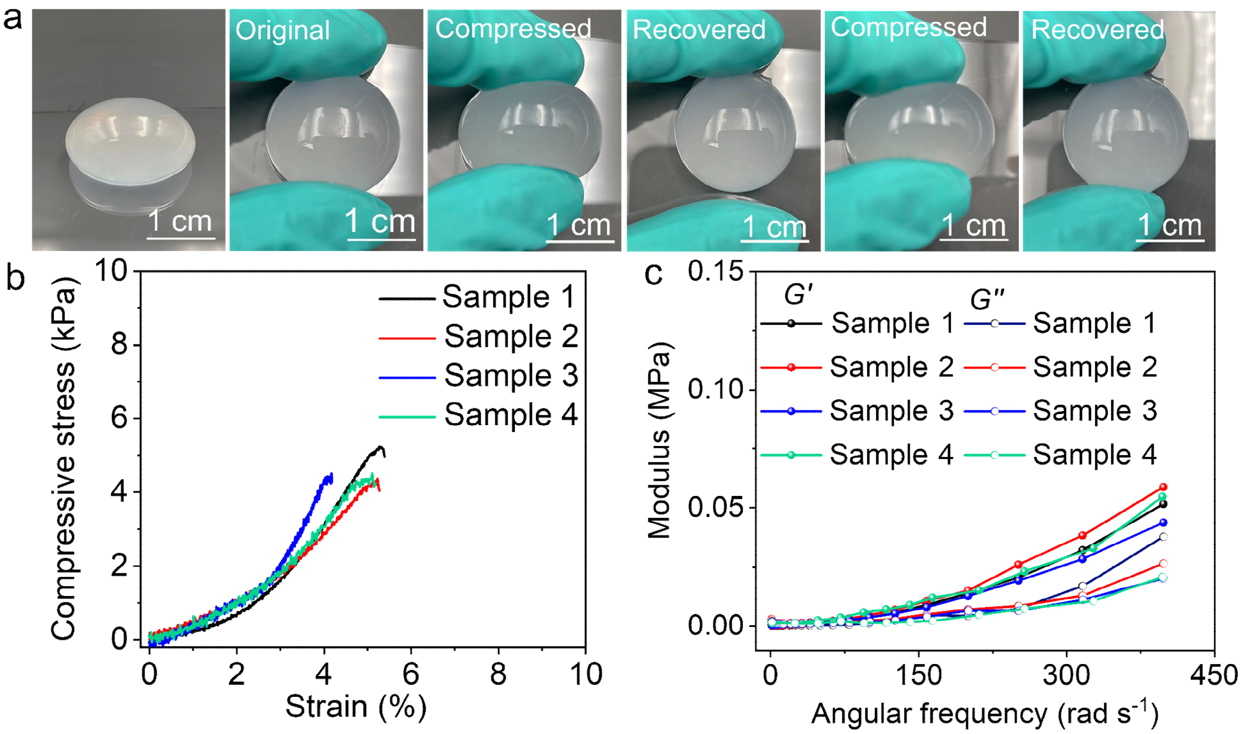

Supplementary Fig. 21 Mechanical properties of 0.28% Ca_2-P_2W_{16} NW-octane gels. a Photographs of the 0.28% Ca_2-P_2W_{16} NWs-octane gel, which was compressed and recovered. **b** Typical compressive stress-strain curves of 0.28% Ca_2-P_2W_{16} NW-octane gels. Element symbols and numbers in the color keys indicate four gel samples with the same mass fraction. **c** Rheological study of gels in the frequency sweep mode for the strain amplitude of 1%.

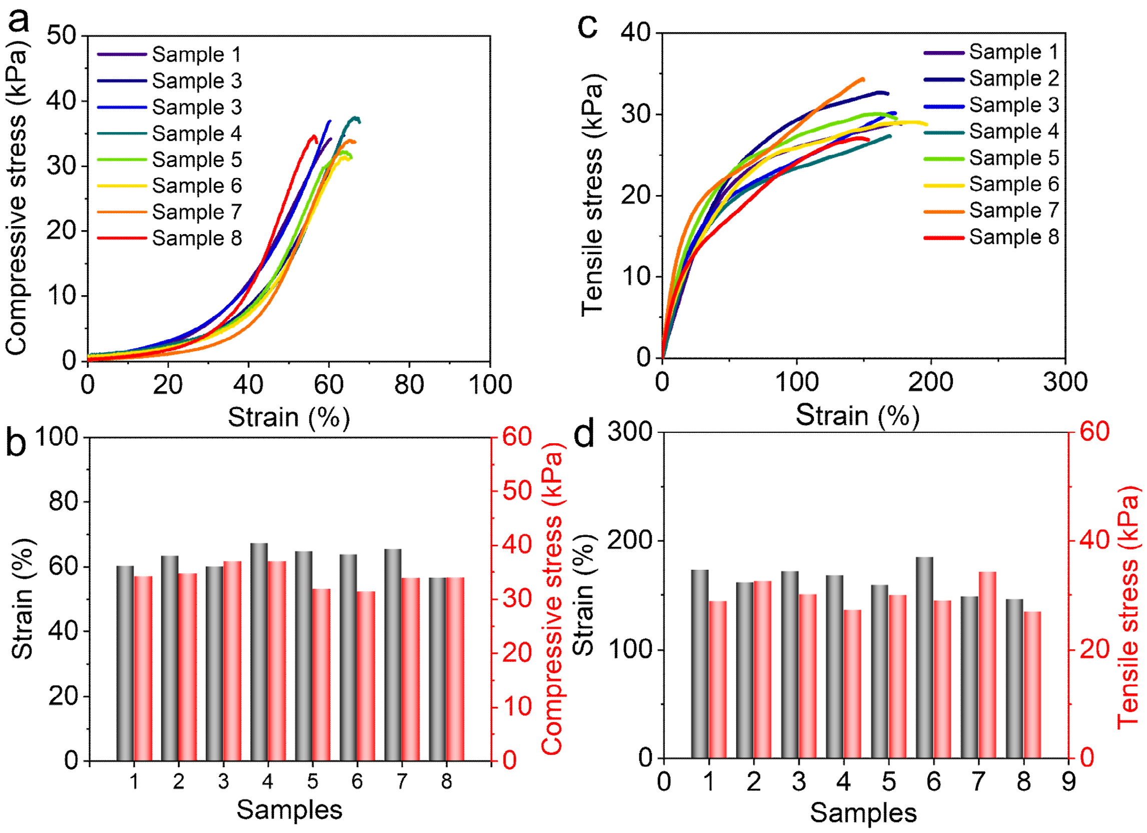

Supplementary Fig. 22 The reproducibility and repeatability of mechanical properties of Ca_2-P_2W_{16} NW-octane gels. a, c Typical compressive stress-strain curves (**a**) and tensile stress-strain curves (**c**) of the gels (10.0%, samples 1-8). **b, d** Histogram of compression strain (%, black bar), compression stress (kPa, red bar) (**b**) and tensile strain (%, black bar), tensile stress (kPa, red bar) (**d**) when eight 1x1x1 cm^3 gels (10.0%, samples 1-8) in the same batch had the maximum strain.

The statistical results showed the reproducibility and repeatability of the mechanical properties testing technique for Ca_2-P_2W_{16} NW-octane gels, with a standard deviation range of less than 10% (Supplementary Table 7).

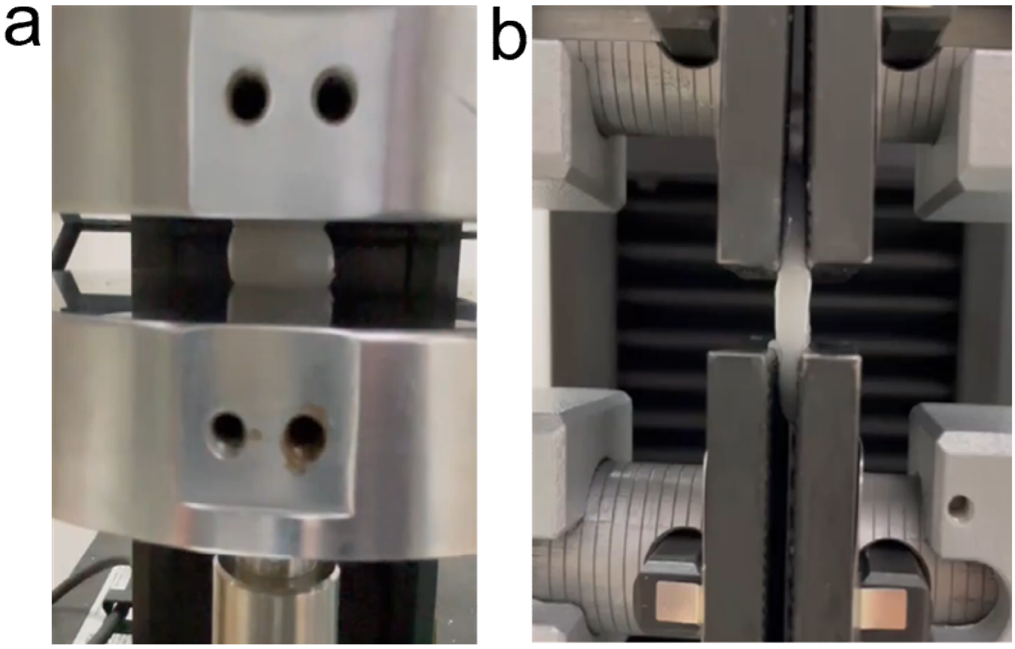

Supplementary Fig. 23 photographs of devices. Compressive load-unload (**a**) and stretch load-unload (**b**) experimental devices of Ca_2-P_2W_{16} NW-octane gels with 10.0% mass fraction of NWs.

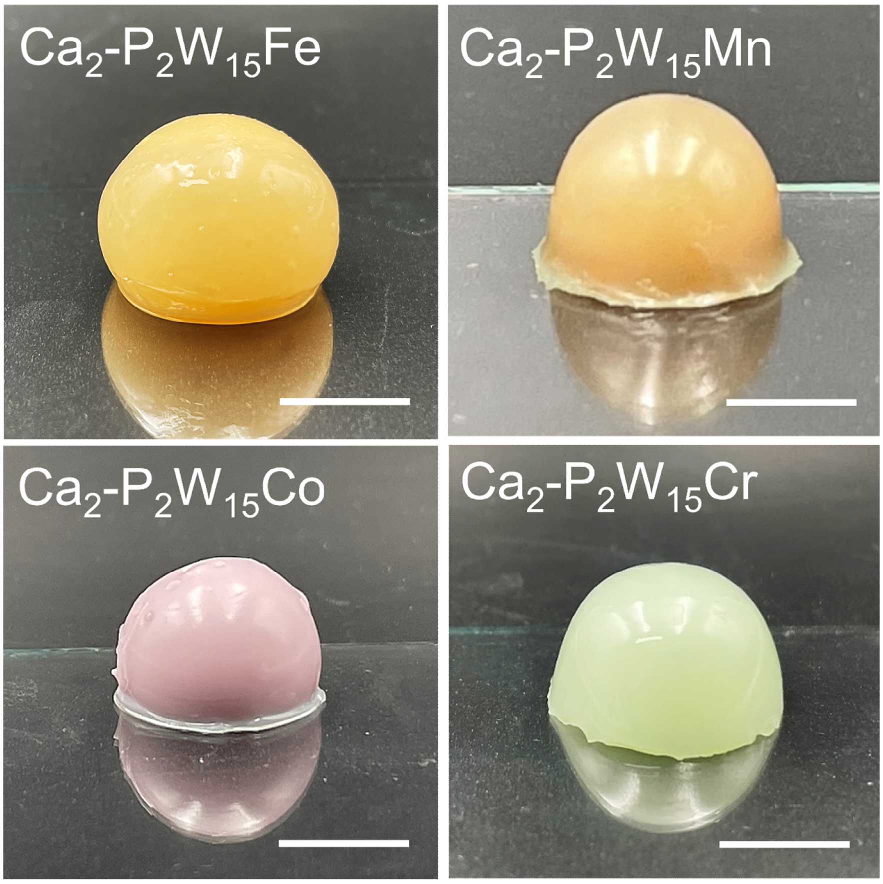

Supplementary Fig. 24 Photographs of Ca_2-$P_2W_{15}M$ (M=Fe, Mn, Co, Cr) NWs-octane gels (10%). Scale bars are 1 cm.

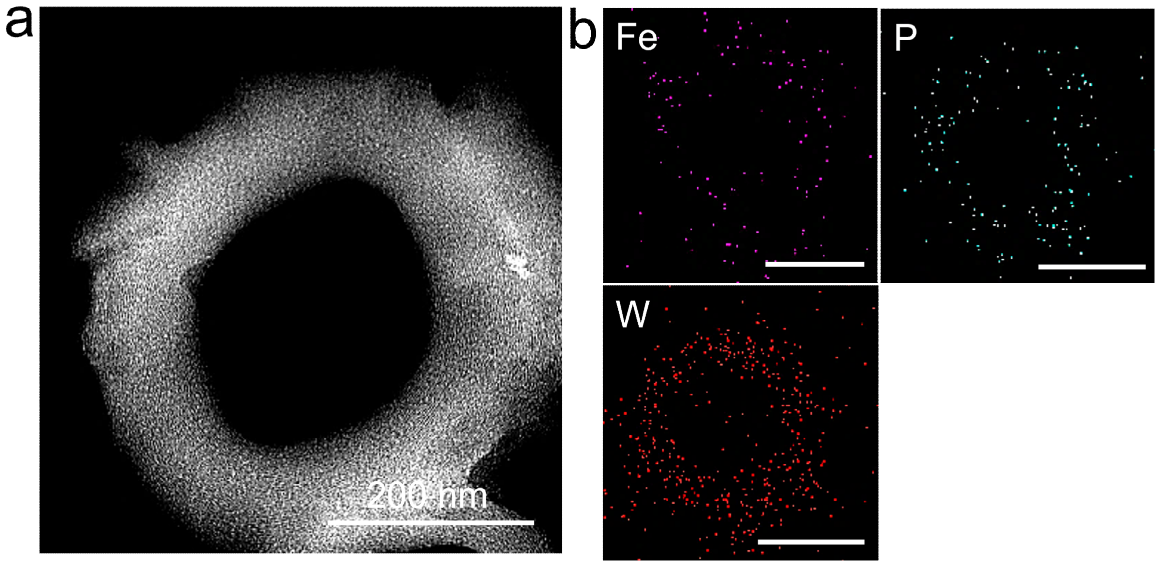

Supplementary Fig. 25 Elemental analysis of Ca_2-$P_2W_{15}Fe$ NWs. STEM image (**a**) and corresponding EDS elemental mapping images (**b**) of coiled Ca_2-$P_2W_{15}Fe$ NWs. Scale bars are 200 nm.

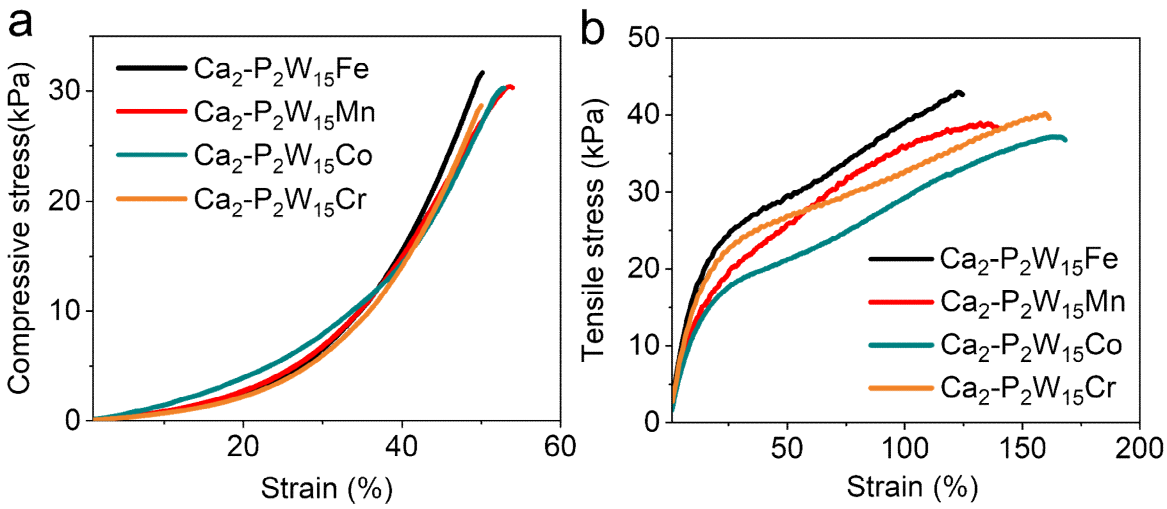

Supplementary Fig. 26 Mechanical properties of Ca_2-$P_2W_{15}M$ (M=Fe, Mn, Co, Cr) NWs-octane gels (10%). The compressive stress-strain curves (**a**) and the tensile stress-strain curves (**b**) of the gels.

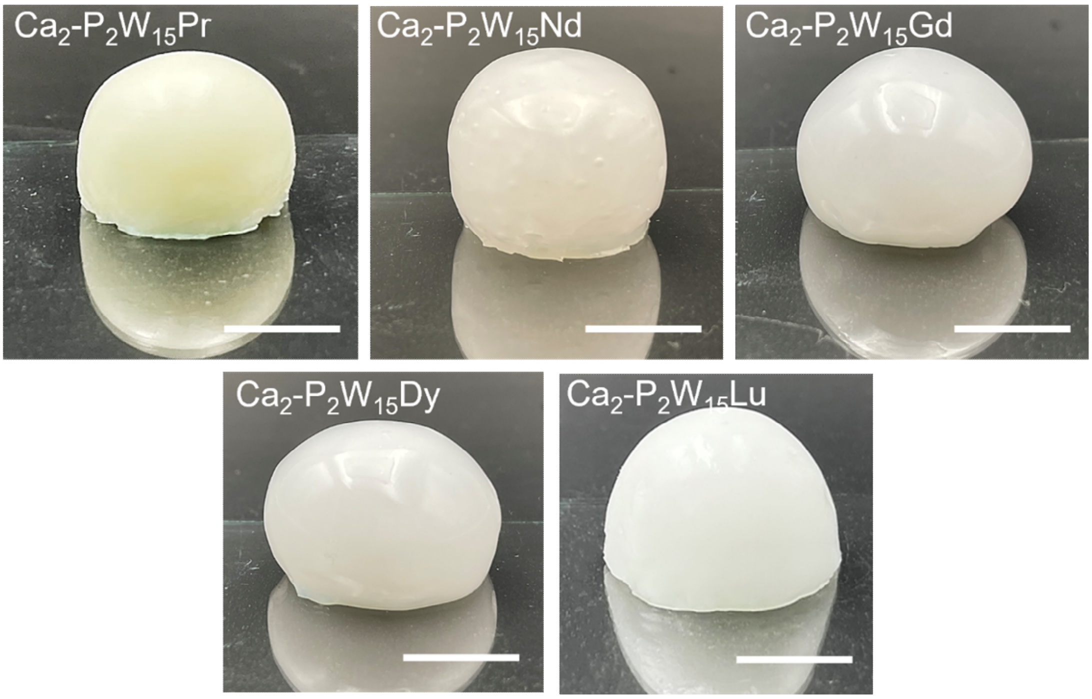

Supplementary Fig. 27 Photographs of Ca_2-$P_2W_{15}M$ (M=Pr, Nd, Gd, Dy, Lu) NWs-octane gels (10%). Scale bars are 1 cm.

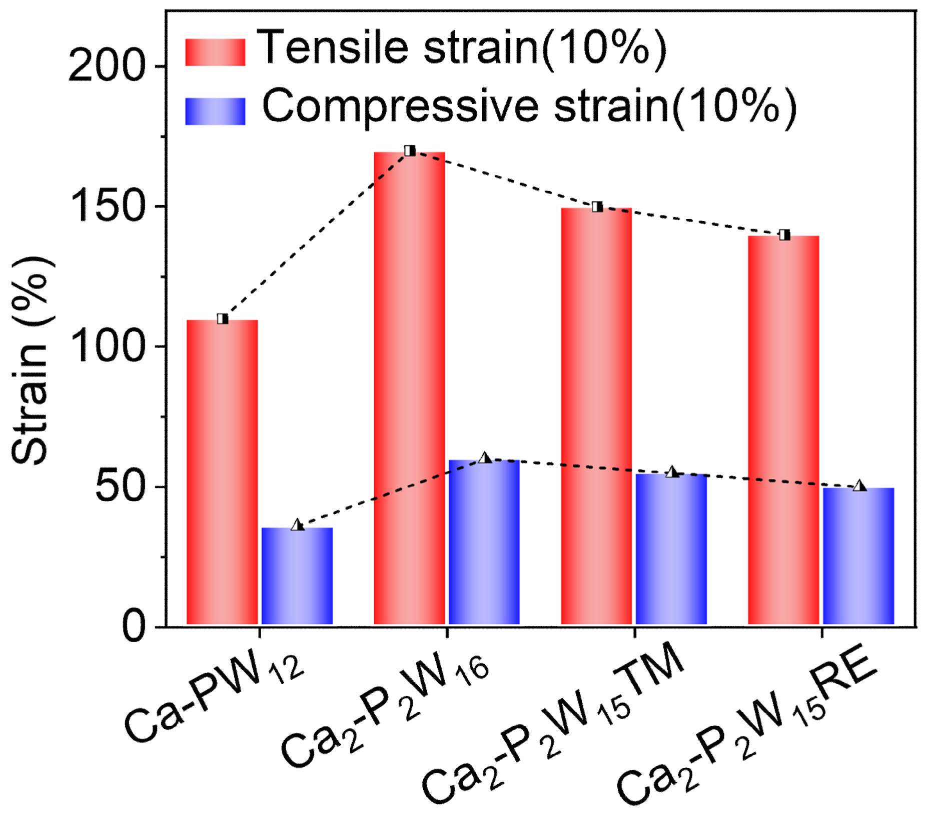

Supplementary Fig. 28 Histogram of compressive and tensile strains of four NWs-based gels (10.0%) at the maximum stress.

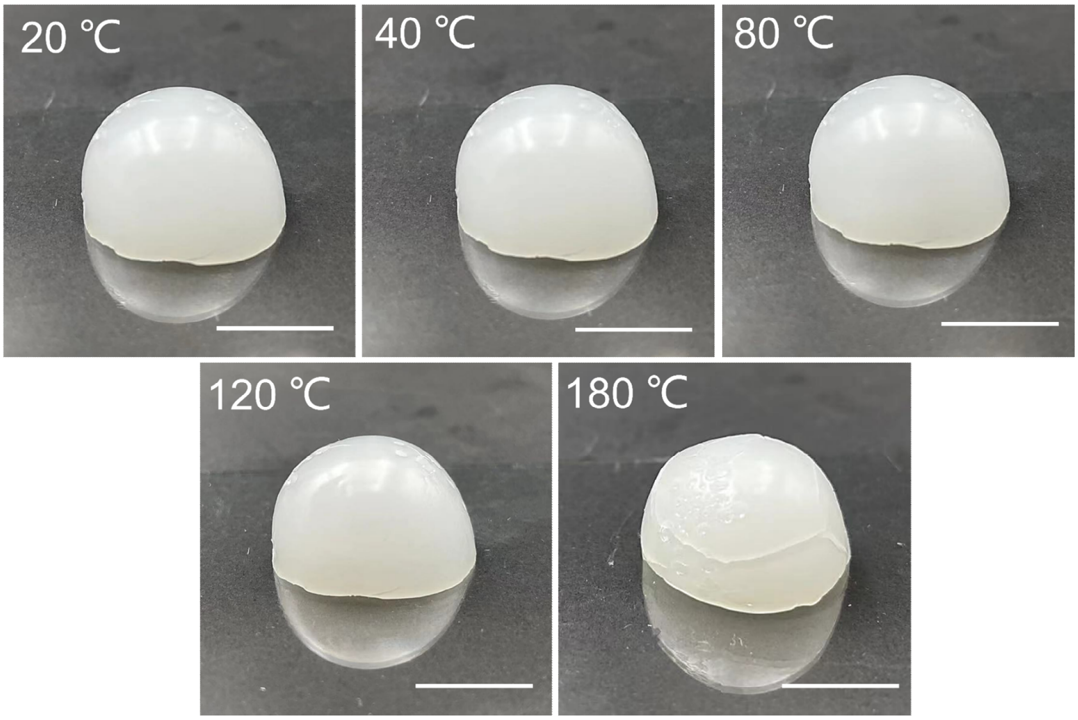

Supplementary Fig. 29 Thermal stability of the Ca_2-P_2W_{16} NW-octadecene gels (10%). Photographs of a gel stay at 20 ℃-180 ℃ for 30 min. The gel can remain stable at temperatures below 120 °C without obvious shrinkage and phase transition. Scale bars are 1 cm.

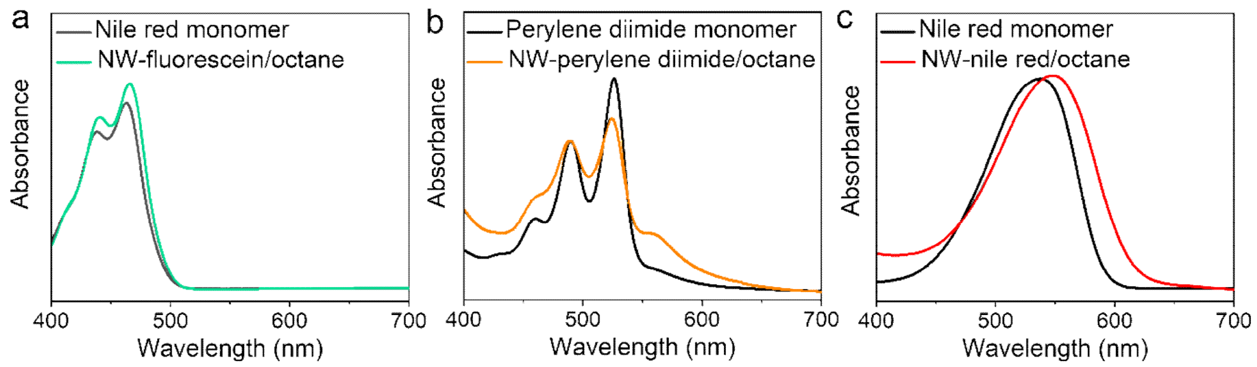

Supplementary Fig. 30 UV-vis spectra of FOM monomers and corresponding Ca_2-P_2W_{16} NW-FOM/octane gels. **a** Ca_2-P_2W_{16} NW-fluorescein/octane. **b** Ca_2-P_2W_{16} NW-perylene diimide/octane. **c** Ca_2-P_2W_{16} NW-nile red/octane.

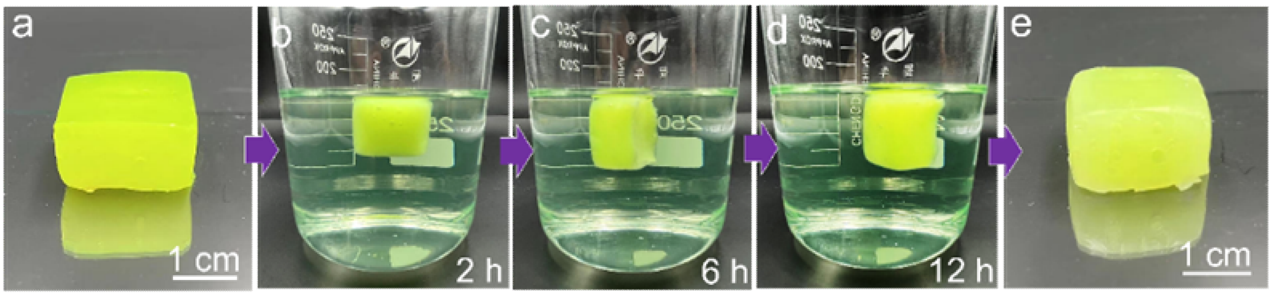

Supplementary Fig. 31 **a** Photograph of Ca_2-P_2W_{16} NW-fluorescein/octane gel (10%). **b-d** Photographs of the gels soaked in octane for 2, 6, and 12 h. **e** Photograph of the gel soaked in octane for 12 h.

3. Supplementary Tables

Supplementary Table 1. The ICP-AES results of Ca_2-P_2W_{16} NWs.

Samples	Atomic ratios
Ca_2-P_2W_{16}	Ca: P: W=1: 1.0: 7.8

Supplementary Table 2. The ICP-AES results of Ca_2-$P_2W_{15}M$ (M=Fe, Mn, Co, Ni, Cr) NWs.

Samples	Atomic ratio
Ca_2-$P_2W_{15}Fe$	Ca: P: W: Fe=1: 0.92: 7.8: 0.48
Ca_2-$P_2W_{15}Mn$	Ca: P: W: Mn=1: 0.89: 7.5: 0.52
Ca_2-$P_2W_{15}Co$	Ca: P: W: Co=1: 0.83: 7.9: 0.45
Ca_2-$P_2W_{15}Ni$	Ca: P: W: Ni=1: 0.85: 7.2: 0.55
Ca_2-$P_2W_{15}Cr$	Ca: P: W: Cr=1: 0.83: 6.8: 0.43

Supplementary Table 3. The ICP-AES results of Ca_2-$P_2W_{15}M$ (M=Pr, Nd, Gd, Dy, Lu) NWs.

Samples	Atomic ratio
Ca_2-$P_2W_{15}Pr$	Ca: P: W: Pr=1: 0.95: 6.8: 0.44
Ca_2-$P_2W_{15}Nd$	Ca: P: W: Nd=1: 0.90: 7.5: 0.42
Ca_2-$P_2W_{15}Gd$	Ca: P: W: Gd=1: 0.93: 7.4: 0.45
Ca_2-$P_2W_{15}Dy$	Ca: P: W: Dy=1: 0.88: 7.1: 0.51
Ca_2-$P_2W_{15}Lu$	Ca: P: W: Lu=1: 0.93: 6.9: 0.43

Supplementary Table 4. The persistence lengths and rupture forces of NWs and other materials

Samples		Persistence length (nm)	Rupture force (pN)
DNA	ss DNA [4]	4	--
	ds DNA [5]	50	--
Covalent bond	Silicon-carbon bond [6]	--	2000±300
	Sulfur-gold anchor [6]	--	1400±300
NWs	GdOOH [7]	< 10	50-200
	BiO-PMA [7]	< 10	50-200
	$Ca\text{-}PW_{12}$ [7]	0.22±0.08[a]	50-200
	$Ca_2\text{-}P_2W_{16}$	1.13±0.31	131.6±21.7
	$Ca_2\text{-}P_2W_{15}Fe$	0.58±0.28	162.5±25.0
	$Ca_2\text{-}P_2W_{15}Mn$	0.53±0.22	150.6±24.3
	$Ca_2\text{-}P_2W_{15}Co$	0.71±0.29	172.2±27.9
	$Ca_2\text{-}P_2W_{15}Ni$	0.79±0.29	143.6±21.9
	$Ca_2\text{-}P_2W_{15}Cr$	0.69±0.29	139.8±19.8
	$Ca_2\text{-}P_2W_{15}Pr$	0.93±0.18	294.3±25.6
	$Ca_2\text{-}P_2W_{15}Nd$	1.08±0.18	253.3±20.8
	$Ca_2\text{-}P_2W_{15}Gd$	1.13±0.20	276.3±24.2
	$Ca_2\text{-}P_2W_{15}Dy$	1.21±0.23	256.5±28.9
	$Ca_2\text{-}P_2W_{15}Lu$	1.12±0.22	284.8±26.3

Supplementary Table 5. Gelation properties of Ca_2-P_2W_{16} NWs in organic liquids

	Solvent	Phase	CGC (%) [a]	Thermal stability range (C°)
Good solvent	n-hexane	Gel	1.22	< 40
	n-heptane	Gel	0.68	< 65
	n-octane	Gel	0.28	< 100
	Octadecene	Gel	0.30	< 120
	Toluene	Gel	1.23	< 75
	Cyclohexane	Gel	1.10	< 45
Bad solvent	Ethanol	Precipitation	---	---
	Formamide	Precipitation	---	---
	Ethylenediamine	Precipitation	---	---
	Acetonitrile	Precipitation	---	---
	Isopropanol	Precipitation	---	---
	Acetone	Precipitation	---	---

[a] CGC: The critical gel concentration (%).

Supplementary Table 6. The yield points of NW-based organogels

Samples		Mass fraction (%)	Yield point (MPa)
Ca_2-P_2W_{16}	Ca_2-P_2W_{16}	10.0%	0.0273
	Ca_2-P_2W_{16}	8.3%	0.0196
	Ca_2-P_2W_{16}	6.2%	0.0122
	Ca_2-P_2W_{16}	3.0%	0.0071
Ca_2-$P_2W_{15}M$ (M=TM)	Ca_2-$P_2W_{15}Fe$	10.0%	0.0264
	Ca_2-$P_2W_{15}Mn$	10.0%	0.0231
	Ca_2-$P_2W_{15}Co$	10.0%	0.0191
	Ca_2-$P_2W_{15}Cr$	10.0%	0.0246
	Ca_2-$P_2W_{15}Cr$	10.0%	0.0241
Ca_2-$P_2W_{15}M$ (M=RE)	Ca_2-$P_2W_{15}Pr$	10.0%	0.0244
	Ca_2-$P_2W_{15}Nd$	10.0%	0.0232
	Ca_2-$P_2W_{15}Gd$	10.0%	0.0229
	Ca_2-$P_2W_{15}Dy$	10.0%	0.0231
	Ca_2-$P_2W_{15}Lu$	10.0%	0.0256

Supplementary Table 7. The tensile strength and compressive strength of Ca_2-P_2W_{16} NW-based organogels

Mass fraction (%)	Tensile strength (kPa)	Compressive strength (kPa)	Number of samples
10.0%	29.0±2.3	34.5±1.7	40
8.3%	23.1±2.1	25.5±2.0	40
6.2%	16.6±1.5	12.5±1.1	40
3.0%	10.5±0.9	4.9±0.3	40

4. Supplementary references

1. Salem, L. Attractive forces between long saturated chains at short distances. *J. Chem. Phys*. **37**, 2100-2113 (1962).

2. DelRio, F. W., Jaye, C., Fischer, D. A. & Cook, R. F. Elastic and adhesive properties of alkanethiol self-assembled monolayers on gold. *Appl. Phys. Lett.* **94**, 131909 (2009).

3. Jia, Y., Zhou. Z., Jiang, H. & Liu, Z. Characterization of fracture toughness and damage zone of double network hydrogels. *J. Mech. Phys. Solids* **169**, 105090 (2022).

4. Tinland, B., Pluen, A., Sturm, J. & Weill, G. Persistence length of single-stranded DNA. *Macromolecules*, **30**, 5763-5765 (1997).

5. Bustamante, C., Marko, J. F., Siggia, E. D. & Smith, S. Entropic elasticity of λ-phage DNA. *Science* **265**, 1599-1600 (1994).

6. Grandbois, M., Beyer, M., Rief, M., Clausen-Schaumann, H. & Gaub, H. E. How strong is a covalent bond? *Science* **283**, 1727-1730 (1999).

7. Shi, Y., Shi, W., Zhang, S. & Wang, X. Revealing the flexibility of inorganic sub-nanowires by single-molecule force spectroscopy. *CCS Chem.* 10.31635/ccschem.023.202302729 (2023).
