# Supplementary material for: Mechanically tunable organogels from highly charged polyoxometalate clusters loaded with fluorescent dyes
Source: Nat Commun. 2023 Dec 14;14:8327. doi: 10.1038/s41467-023-43989-3 (PMC10721816; doi:10.1038/s41467-023-43989-3)
Supplement: Supplementary file 3 — Description of Additional Supplementary Files [file 41467_2023_43989_MOESM3_ESM.pdf]

Description of Additional Supplementary Files

File Name: Supplementary Movie 1
Description: The mechanical properties of Ca_2-P_2W_{16} NW-octane gels under pressure were characterized using a universal test machine (Instron 3400). The load-unload cycle test to obtain 100 times compressive stress-strain curves of gels with a 10.0% mass fraction of Ca_2-P_2W_{16} NWs. (The movie is 20 times faster than the original video)

File Name: Supplementary Movie 2
Description: The mechanical properties of Ca_2-P_2W_{16} NW-octane gels under stretch were characterized using a universal test machine (Instron 3400). The load-unload cycle test to obtain 100 times tensile stress-strain curves of gels with a 10.0% mass fraction of Ca_2-P_2W_{16} NWs. (The movie is 20 times faster than the original video)

File Name: Supplementary Movie 3
Description: The mechanical properties of Ca_2-P_2W_{16} NW-octane/FOM gels (10.0%) under pressure.
